# Photocatalytic Degradation of Aqueous Rhodamine 6G Using Supported TiO_2_ Catalysts. A Model for the Removal of Organic Contaminants From Aqueous Samples

**DOI:** 10.3389/fchem.2020.00365

**Published:** 2020-05-05

**Authors:** Eduardo Pino, Cristian Calderón, Francisco Herrera, Gerardo Cifuentes, Gisselle Arteaga

**Affiliations:** ^1^Facultad de Química y Biología, Universidad de Santiago de Chile, Santiago, Chile; ^2^Departamento de Ingeniería Metalúrgica, Universidad de Santiago de Chile, Santiago, Chile; ^3^Departamento de Ingeniería Química, Universidad de Santiago de Chile, Santiago, Chile

**Keywords:** photocatalytic degradation, organic dyes, water treatment, contaminant, semiconductor sensitizer

## Abstract

As a model for the removal of complex organic contaminants from industrial water effluents, the heterogeneous photocatalytic degradation of Rhodamin 6G was studied using TiO_2_-derived catalysts, incorporated in water as suspension as well as supported in raschig rings. UV and Visible light were tested for the photo-degradation process. TiO_2_ catalysts were synthesized following acid synthesis methodology and compared against commercial TiO_2_ catalyst samples (Degussa P25 and Anatase). The bandgap (E_g_) of the TiO_2_ catalysts was determined, were values of 2.97 and 2.98 eV were obtained for the material obtained using acid and basic conditions, respectively, and 3.02 eV for Degussa P25 and 3.18 eV for anatase commercial TiO_2_ samples. Raschig rings-supported TiO_2_ catalysts display a good photocatalytic performance when compared to equivalent amounts of TiO_2_ in aqueous suspension, even though a large surface area of TiO_2_ material is lost upon support. This is particularly evident by taking into account that the characteristics (XRD, RD, Eg) and observed photodegradative performance of the synthesized catalysts are in good agreement with the commercial TiO_2_ samples, and that the RH6G photodegradation differences observed with the light sources considered are minimal in the presence of TiO_2_ catalysts. The presence of additives induce changes in the kinetics and efficiency of the TiO_2_-catalyzed photodegradation of Rh6G, particularly when white light is used in the process, pointing toward a complex phenomenon, however the stability of the supported photocatalytic systems is acceptable in the presence of the studied additives. In line with this, the magnitude of the chemical oxygen demand, indicates that, besides the different complex photophysical processes taking place, the endproducts of the considered photocatalytic systems appears to be similar.

## Introduction

The removal of hazardous organic contaminants derived from human productive activities, present in the environment and particularly in water sources has become an important research topic aimed toward the development of sustainable water treatment strategies and processes.

The leather, paper, plastic and textile industries use dyes to color their products while using large volumes of water (Robinson et al., 2001; Yaseen and Scholz, 2019), with more than 10,000 types of commercial dyes and 70,000 tons of waste are produced annually. This discharge of wastewater to natural streams leads to major problems, such as an increase in toxicity and oxygen demand of the effluents, as well as a reduction of the amount of light that can pass through the water, producing a negative effect on the phenomena of photosynthesis of aquatic life. The color of wastewater is the first public perception of contamination, the presence of small amounts of dyes (1 ppm) is highly visible and undesirable, due to their high molar absorptivity coefficients.

For the vast array of wastewater treatment technologies currently in use, namely adsorption on activated carbon (Foo and Hameed, 2009), ultrafiltration, coagulation by chemical agents and resins of synthetic adsorbents, biological treatment, electrocatalytic decomposition (Fujishima and Honda, 1972; Daghrir et al., 2012), etc., factors such as the sheer complexity of the organic contaminants found in waste water effluents call for a simpler yet transversal solution, able to yield a proper removal of the contaminants by an aggressive oxidative decomposition. From this, advanced oxidation processes (AOPs) for chemical degradation have become simple and effective methods for the elimination of organic contaminants (Giménez et al., 2015).

Several research groups have sought to optimize the process for the degradation of organic pollutants in water (Rizzo et al., 2009; Chong et al., 2010), so that it meets the requirements of efficiency, easy to handle, and improved time of degradation, by allowing the pollutants complete mineralization, that is, the formation of carbon dioxide (CO_2_), water (H_2_O) and other inorganic compounds such as HCl, HNO_3_, etc. and/or the generation of less toxic organic byproducts that are environmentally safe (Amenn et al., 2013). Based on this premise, photocatalytic degradation has become a widespread subject of study, focused on making use of the particular interactions that takes place between light and semiconductive materials (SCM), in a process termed as heterogeneous photocatalysis (Ahmed et al., 2011; Teoh et al., 2012), that allows the degradation of organic molecules via advanced oxidative pathways, due to the abundant generation of radicals on the surface of the SCM by electronic excitation elicited by the incident light.

Heterogeneous photocatalysis, has become an efficient alternative to achieve the degradation of many pollutants. This technique uses radiant energy, visible and / or ultraviolet light coming from artificial light sources or directly from the sun, which, upon interacting with a catalyst (semiconductor) (Smith and Nie, 2009), generates a charge separation by means of charge transfer processes, leading to the formation of reactive oxygen species (hydroxyl radicals, superoxide anion, hydrogen peroxide, etc.), necessary for the oxidation and subsequent mineralization of the organic contaminants (Módenes et al., 2012).

One of the most efficient SCM, in terms of both cost and photocatalytic properties, is Titanium dioxide (TiO_2_) with a large number of works published elsewhere (Ajmal et al., 2014; Gaya, 2014), devoted to in-deep descriptions of the interesting photophysical properties of this material. Briefly, the ability of SCM such as TiO_2_ to be activated by photon absorption is associated to the energy difference, or bandgap (Eg) that separates their valence band (VB) electrons from their counterparts in the conduction band (CB), where Eg is usually lower than 5 eV in common SCM (Smith and Nie, 2009). Upon light excitation, electrons move from VB to CB, leaving a hole (h^+^) into the VB. Further, the transferred electrons can participate in the generation of reactive oxygen species (ROS), leading to occurrence of photocatalytic degradation. A general description of the photocatalytic degradation of dyes in the presence of TiO_2_ is shown in Equations (1–6).

(1) $$\begin{array}{l} TiO_{2}+h\nu \;\left(UV\right)\to TiO_{2}\left(e_{CB}^{-}+h_{VB}^{+}\right) \end{array}$$

(2) $$\begin{array}{l} TiO_{2}\left(h_{VB}^{+}\right)+H_{2}O\;\to TiO_{2}+H^{+}+OH^{∙} \end{array}$$

(3) $$\begin{array}{l} {\;TiO}_{2}\left(h_{VB}^{+}\right)+HO^{-}\;\to TiO_{2}+OH^{∙} \end{array}$$

(4) $$\begin{array}{l} {\;TiO}_{2}\left(e_{CB}^{-}\right)+O_{2}\;\to TiO_{2}+O_{2}^{∙-} \end{array}$$

(5) $$\begin{array}{l} O_{2}^{∙-}+H^{+}\to HO_{2}^{∙}\to H_{2}O_{2}\;\to OH^{∙} \end{array}$$

(6) $$\begin{array}{l} Dye+ROS\to Products \end{array}$$

TiO_2_ has three crystalline phases, anatase (tetragonal), rutile (octahedral) and brookite (orthorhombic) (Landmann et al., 2012), with anatase being the most active photocatalytically due to the combined effect of a lower recombination rate and a higher capacity of adsorption on the surface (Carreon et al., 2011). The TiO_2_ in mixed phase have a greater photocatalytic activity compared to a pure crystalline phase (Hurum et al., 2003). TiO_2_ use is limited by fact that the absorption of light corresponding to the Eg for the cristalline forms of TiO_2_ falls in the ultraviolet range, which is one of the minority components of the solar spectrum. This disadvantage of the semiconductor is due to its high value of Eg, being 3.2 eV for anatase and 3.02 eV for rutile. To extend the range of absorption of the catalysts to the visible spectrum and decrease the recombination of the pair (${\text{h}}_{\text{VB}}^{+}$ / ${\text{e}}_{\text{CB}}^{-}$) _one_ of the strategies used has been the use of photosensitizing dyes (Stracke and Heupel, 1999). Upon irradiation, the transfer of electrons from the excited state of the dye to the conduction band of the semiconductor can be produced, in a process defined as “sensitization” (equations 7-19) of the TiO_2_ (Wu et al., 1998). This process only will be possible if the energy level of the excited state of the dye (Dye^*^) is higher than the energy level of the conduction band. The injected electrons can be transferred to the oxygen adsorbed on the TiO_2_ surface to form superoxide anion radicals, which lead to the formation of ROS (Dyi-Hwa et al., 2012), these species being responsible for the oxidation of organic matter (Song et al., 2016). The sensitizing dye is also degraded in the process (equations 5 and 6), making this synergy between the sensitizing dye and TiO_2_, ideal for the decomposition of organic contaminants able to sensitize TiO_2_ using white light, minimizing the use of high energy radiation (ca. UV light) by ignoring the Eg value of the photocatalyst.

(7) $$Dye+hv\to \;Dye^{*}$$

(8) $$Dye^{*}\to \;bleaching\;or\;photophysical\;process$$

(9) $$TiO_{2}+Dye^{*}\left(e^{-}\right)\to TiO_{2}\left(e_{cb}^{-}\right)+Dye^{∙+}\to Products$$

Besides of taking advantage of the dye-sensitizing of TiO_2_, and in order to further extend the applications and usability of TiO_2_ catalysts, the support of the photocatalytic material have become an interesting venture for catalysis research. Current developments for the use TiO_2_ are focused on the support of the photocatalyst on a wide variety of materials (Ansón-Casaos et al., 2013; Ranjith et al., 2019) which may set the basis for the implementation of applied waste-water treatment solutions. Polymeric supports have been considered to enhance and control the photocatalytic properties of TiO_2_. Recently, poly (ethyleneterephtalate)-supported TiO_2_ composite films (Malesic-Eleftheriadou et al., 2019) have proven useful for the photocatalytic degradation of complex antibiotic mixtures irradiated with simulated solar light, leading to high photocatalytic efficiencies and good reusability even when low content (10%) of supported TiO_2_ is used. Hyerarchical wrinkled mesoporous silica used as support for TiO_2_ catalysts (Wan et al., 2018), have shown high TiO_2_ support yield and enhanced photodegradation activity toward organic dyes, particularly the TiO_2_ catalyzed photodegradation of Rhodamin B under UV light exposition, where the performance of the supported catalyst can be modulated by controlling the calcination temperature during the TiO_2_ support step.

Studies evaluating the performance of metal oxide nanoparticles/TiO2 heterojunctions have reported an enhanced degradation of Rhodamine B. For example, ZnO/TiO_2_ heterojunction photocatalysts (Wang et al., 2018), in a degradation process mediated by the formation of a direct Z-scheme heterojunction structure formed between ZnO and TiO_2_, with hydroxyl and superoxide anion radical playin relevant roles in the phtocatalytic process. Similarly, enhanced photocatalytic performance under visible light irradiation have been observed for p-n heterojunctions formed in TiO_2_ nanofibers decorated with Ag_2_O nanoparticles (Liu et al., 2019), effect mainly attributed to the fast separation of the photogenerated electron-hole pairs and high light absorption efficiency of the fibers. On the other hand, nanophotocatalysts based on TiO_2_/SrTiO_3_ heterojunctions supported on activated carbon (Ali et al., 2019) have displayed exceptional activities, compared to commercial TiO_2_ samples, on the photodegradation of pollutants such as 2,4-dichlorophenol and bisphenol A, where the presence of the activated carbon allows the enhancement of the photocatalystic activity by increasing the adsorption of O_2_, as well as by accepting the electrons from the semiconductors heterojunction.

Degussa-P25 TiO_2_ catalyst supported on mullite ceramic foam was tested in a photocatalytic ozonization process for the degradation of N-N-diethyl-m-toluamide (Rodríguez et al., 2019). The performance of the mullite supported catalysts was close to that observed when raschig rings where used as support, where the combination of the ozone and TiO_2_ photocatalyzed degradation had a negative impact on the degradation rate, but higher efficiency on the mineralization process of the substrate.). TiO_2_ supported in activated carbon has also been used for the photocatalytic decomposition of the micotoxin aflatoxin B1 (Sun et al., 2019), a carcinogen agent that can be found in vegetal and animal feedstock, with good performance of the photocatalyst when UV-Vis light is used for irradiation, with enhanced photodegradation of the supported material when compared with the bare photocatalysts. Similarly, hybrid TiO_2_ catalysts supported on reduced graphene oxide (Ranjith et al., 2019) displayed good performance in the oxidative degradation of organic dyes (methylene blue and crystal violet) by irradiation with visible light, with the dye degradation taking place through electron-hole separation.

Beyond the development of TiO_2_ supported in microgranular porous materials, larger structures of supporting material have been less explored. For example, periodic and flow reactors using TiO_2_ catalysts in suspension as well as supported on glass fabric have been used for the UV-Vis photocatalyzed degradation of sertraline, an antidepressant drug, from aqueous samples (Rejek and Grzechulska-Damszel, 2018). The photodegradation yield where highly dependent on the configuration of photoreactor, where the highest degradation percentages where achieved using the periodic reactor containing the TiO_2_-coated glass fabric. TiO_2_ coated natural and synthetic non-woven fibers have also been tested on the photocatalyzed degradation of the textile dye reactive yellow 145 (Alahiane et al., 2014) where good degradation performance under irradiation with UV light, was achieved under several conditions, such as the presence of additives, namely ethanol, hydrogen peroxide, inorganic anions, as well as optimal degradation in acidic media (pH = 3).

The present work will focus on the study of TiO_2_ catalysts supported on borosilicate glass rings (raschig rings), which will allow the development and optimization of a photocatalytic degradation process based on the use of both UV and white light, taking advantage of the ability of the different crystal structures of TiO_2_, leading to a controllable photodegradation process of complex organic molecules by control of the free radical generation process on the supported TiO_2_ catalysts, either by the energy of the incident light or the combination of different TiO_2_ crystal structures, allocated on the vitreous support. The physically and chemically stable supported photocatalytic structures will yield reusable materials for the implementation of water decontamination strategies either for batch or continuous regime water treatment, providing stability to the TiO_2_ particles, enhancement of the catalytic surface to the incident light as well as adsorption of the substrates for the degradation, combined with good mass transport through the material, by taking advantage of the intrinsic properties of the design of the raschig rings as packing material, for example, in engineering application of fractionation columns (Raja et al., 2005).

Due to the fact that colored dyes, commonly found as waste water organic contaminants usually share similarities in their structures, a model compound is required in order to test the proposed TiO_2_ catalysts in a streamlined and proper fashion (Lasio et al., 2013; Bokhale et al., 2014), allowing further analysis and interpretation of the obtained results. In this context, xanthenic dyes stands out as a suitable candidates. Rhodamine 6G (Rh6G) also known as Rhodamine 590, belongs to the xanthenes family, which are largely used to synthesize drugs and to prepare dyes of the fluorescein and eosin class. Rhodamine 6G is a cationic polar dye with a rigid heterocyclic structure, which exhibits a strong absorption in the visible and an intense fluorescence (Magde et al., 1999; Bujdak and Iyi, 2012; Zehentbauer et al., 2014). Rh6G is widely used in acrylic, nylon, silk, wool and dyeing, it is the dye most used for dye laser applications and as a fluorescent tracer to visualize flow patterns as for example in the field of hydraulics (Tarud et al., 2010). Rh6G is commonly used as a sensitizer (Wu et al., 1998). In recent years, a growing number of studies have attempted to incorporate Rh6G into inorganic and organic matrices (Vanamudan and Pamidimukkala, 2015) for application in fields such as solid-state laser action, optoelectronics and optical filters, among others (Barranco and Groening, 2006).

Our analysis comprises the use of TiO_2_ catalysts synthesized by a sol-gel methodology, as well as commercial samples of TiO_2_ (Anatase and Degussa P25) supported in raschig rings and the photocatalytic activity of the supported catalysts evaluated by monitoring the degradation of Rh6G under irradiation with UV (365 nm) and white light (400–700 nm) light sources. Further, the influence of additives that can be usually found accompanying organic dyes in waste water, such as sulfates and chlorides (Guillard et al., 2005), as well as photocatalysis promoters such as hydrogen peroxide (Li et al., 2001), will be evaluated in our photocatalytic systems.

## Experimental

### Materials

Nitric Acid, titanium dioxide (Anatase), titanium dioxide (Degussa P25), titanium isopropoxide, polyethylene graft maleic anhydride and Rhodamin 6G were purchased from Sigma-Aldrich. Sodium chloride, n-hexane, sodium hydroxide, hydrogen peroxide and sodium sulfate were purchased from Merck. All reagents were used as received.

### Methods

#### Synthesis of TiO_2_

The synthesis of TiO_2_ was performed by the Sol-gel method (Ochoa et al., 2009). A mixture of 100 mL of ultrapure water and 27 mL of isopropanol was used to dissolve 16.6 ml of titanium isopropoxide (Mahshid et al., 2007), under constant stirring for 20 min. Later, depending of the acidity required, 3.1 mL HNO_3_ 0.032 M (acid synthesis) were added and the suspension kept under constant stirring at 80°C for 20 hrs. Finally, the resulting gel was treated in a muffle furnace for 4 h at 560°C and then left to cool at room temperature to recover the solid TiO_2_.

#### TiO_2_ Support on Raschig Rings

In a crystallizer containing 0.25 g of polyethylene graft maleic anhydride (PEGMA), completely dissolved in 25 mL of hexane, 50 Raschig rings were incorporated and the temperature raised up to 70°C to achieve total evaporation of the solvent. The dried rings were added to an aqueous TiO_2_ suspension (10 g/L for the synthesized TiO_2_ catalysts or 1 g/L for the commercial TiO_2_ samples) and left to rest for 30 min. Later, the solvent was evaporated by heating the suspension at 150°C followed by elimination of residual organic matter by heating at 500°C for 2 h in a muffle furnace (Raja et al., 2005). The TiO_2_ support efficiency was calculated by determining the amount of TiO_2_ supported on the rings (the total amount of rings used on the support step) by weight differences, and the resulting mass of TiO_2_ supported was expressed as a percentage in relation to the total mass of TiO_2_ used in the support step.

#### Characterization of the TiO_2_ Catalysts

X-ray diffraction analysis of the synthesized TiO_2_ catalysts were performed in order to discriminate the crystal structures present in the resulting material, and the results were compared with commercial TiO2 samples. XRD data was obtained using a Shimadzu XRD-6000 (Cu, Kα, Ni Filter, 40 kV, 30 mA) difractometer, with a 2 min^−1^ scan speed. Scanning electronic microcopy (SEM) analysis of the samples was performed in a JEOL JSM-7800F scanning electron microscope equipped wtih a X-ACT Cambridge instruments detector for energy dispersive X-ray (EDX) analysis.

BET adsorption isotherms and specific surface area of the studied TiO_2_ catalysts were determined by using a Micromeritics ASAP 2020 systing, with N_2_ gas as adsorbate at 77K.

The bandgap energy (Eg) of the synthesized TiO_2_ catalysts was determined by diffuse reflectance experiments (López and Gómez, 2012), using the Kubelka-Munk function (FKM) for the analysis of the diffuse reflectance (R) of TiO_2_, according to Equations (1, 2).

(1) $$\begin{array}{l} R=\;\frac{R_{sample}}{R_{reference}} \end{array}$$

(2) $$\begin{array}{l} FKM=F\left(R\right)=\frac{{\left(1-R\right)}^{2}}{2R} \end{array}$$

The Tauc mathematical model was used for the accurate determination of the Eg values. Briefly, this model proposes that, for materials with a direct band gap [REF], the magnitude of Eg can be estimated by Equation 3.

(3) $$\begin{array}{l} \alpha h\nu =A{\left(h\upsilon -E_{g}\right)}^{n} \end{array}$$

Where ν is the frequency of the incident light, A correspond to a proportionality constant and α is a linear absorption coefficient. For TiO_2_ and other materials with direct band gap, n is equal to 2 (Smith and Nie, 2009; López and Gómez, 2012). Under specific conditions, the absorption coefficient α is proportional to the FKM, according to Equation 4.

(4) $$\begin{array}{l} F\left(R\right)h\nu =A{\left(h\upsilon -E_{g}\right)}^{n} \end{array}$$

From plots of *F*(*R*)*hν*^1/2^ vs. *hυ*, the linear extrapolation at *F*(*R*)*hν*^1/2^ = 0 allows the determination of Eg.

#### Photodegradation Kinetics of Rhodamin 6G

##### Photostability of Rhodamin 6G

To evaluate the photostability of Rh6G in the absence of catalyst, from a 1.15 mM stock solution of Rh6G, 5 uM Rh6G solutions were freshly prepared and irradiated for 90 min in a Solsim-Luzchem photoreactor using either UV lamps (8 Hitachi FL8BL-B lamps, 365 nm, 8W rated power consumption) or white light lamps (8 Westinghouse 4000K white light T5BF-840 lamps, 8W rated power consumption). Aliquots of the samples were taken every 10 min and their absorbance was measured at 526 nm in an Agilent 8453 UV-Vis spectrophotometer. The same procedure was performed incorporating additives (H_2_O_2_, NaCl, Na_2_SO_4_) to the Rh6G solutions. All the solutions were prepared using ultrapure water and the determinations were performed by triplicate unless otherwise indicated.

For the Raschig rings-supported TiO_2_ catalysts, the photodegradation procedure involve the addition of 10 catalyst-coated Raschig rings to the Rh6G solutions, and keeping the same irradiation protocol previously described for the samples in homogenenous media.

Degradation of Rh6G in aqueous suspensions of TiO_2_ took place under constant irradiation for 30 min. For comparison sake, the amount of TiO_2_ used in the studied suspensions was determined by comparison with the mass of supported catalysts in the experiments involving the Raschig rings.

All the degradation kinetic data was adjusted to a pseudo-first order kinetic model, according to the Langmuir-Hinshelwood kinetic model for reactions taking place in heterogeneous media (Loghambal et al., 2018). All of the kinetics were performed under constant air bubbling. The reported data correspond to the average of at least three independent determinations, unless otherwise stated.

Evaluation of the chemical oxygen demand for the photodegradation of Rhodamin 6G.

The determination of the chemical oxygen demand was performed over the remainder organic matter left after a short photocatalytic degradation course (ca. 10 min). To a tube containing K_2_Cr_2_O_7_, sulfuric acid and silver as a catalyst, 1 mL of a centrifuged solution of Rh6G previously subjected to photodegradation were added. The sample tubes were subjected to digestion at 150°C for 2 hrs. Once the samples are cooled, the concentration of Cr^+3^ was determined by spectrophotometric analysis, measuring the absorbance of the samples at 620 nm (Lenore et al., 2009). Reported results correspond to samples (*n* = 3) measured by triplicate, where the results were deemed suitable when their standard error were under 10% of the average value determined.

## Results and Discussion

### TiO_2_ Catalysts Characterization

**Scheme 1:**
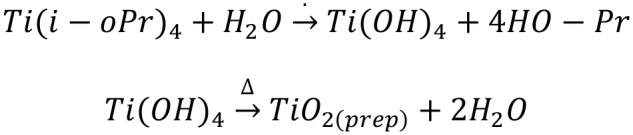
Chemical reactions involved in the synthesis of the studied TiO_2_ catalysts.

TiO_2_ catalysts were synthesized by a Sol-Gel method, starting by the hydrolysis of the metallic alcoxide, followed by calcination of the resulting gel (Scheme 1). The hydrolytic step was performed in acid media, resulting in 90% yield for the reaction.

$$\begin{array}{r} {Ti\left(i-oPr\right)}_{4}+H_{2}O{Ti\left(OH\right)}_{4}+4HO-Pr\\ {Ti\left(OH\right)}_{4}TiO_{2\left(prep\right)}+{2H}_{2}O \end{array}$$

**Figure 1:**
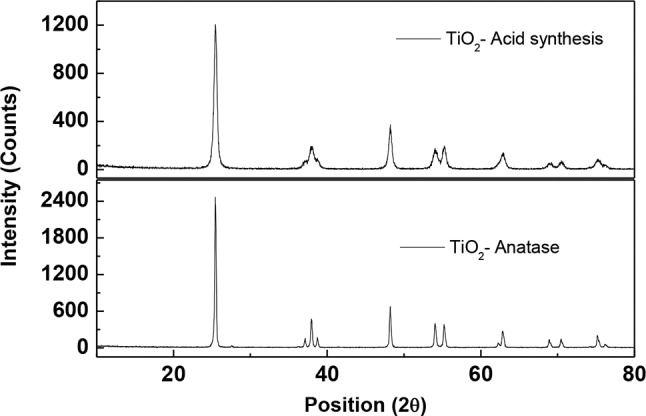
X-ray powder diffraction pattern for the studied TiO_2_ catalysts.

X-ray diffraction analysis (Figure 1) of the synthesized TiO_2_ catalysts herein named as acid synthesis TiO_2_ (AS-TiO_2_), show identical spatial features for the crystal structures of AS-TiO_2_ and Anatase, with signals for AS-TiO_2_ at 2θ values equals to: 25.4; 37.2; 37.9; 38.7; 48.2; 54.2; 55.3; 62.9; 69.0; 70.5; 74.1; 75.2, and 76.2. For Anatase is also observed the presence of minor signals (at values of 2θ equal to 27.6; 36.2 and 41.4), attributed to the presence of traces of the Rutil crystalline form of TiO_2_, which are not observed in the XRD pattern of AS-TiO_2_. The Rutil content in the commercial sample of Anatase is estimated as lower than 2%, according to calculations performed by using the relationship between the respective TiO_2_ phase and the strongest reflection associated to each phase under consideration, according to: *phase %* = *100/(1*+*1.265I*_*R*_*/I*_*A*_*)* (Spurr and Myers, 1957), where I_R_ and I_A_ are the respective intensities of the Anatase and Rutil signals at 2θ values of 25.1 and 27.6, respectively. For the Degussa P25 (DP25) samples, more intense signals at 2θ positions equivalent to those of Rutil are observed, associated with increased content of the Rutil crystalline phase in DP25, material comprised by the Anatase and Rutil phases of TiO_2_ in an 85:15 ratio (See Supplementary Figure 1 in the electronic supplementary information of this work, for the DP25 XRD pattern).

**Figure 2:**
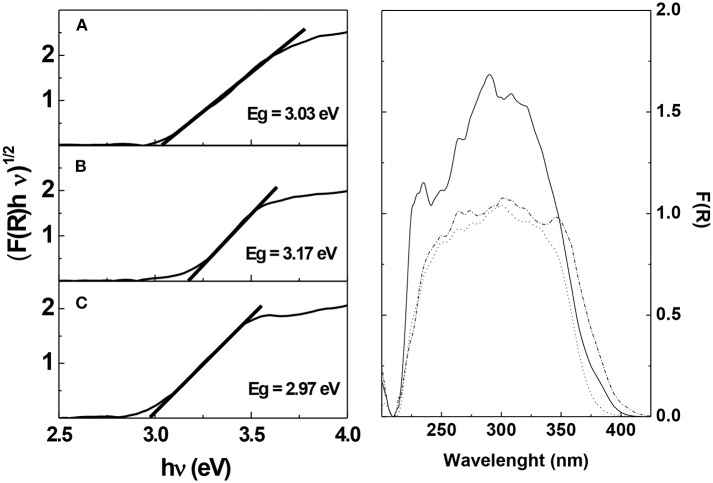
(**Left**) Product between the diffuse refractance and the energy of the incident light represented against the energy of the incident light, according to the Tauc-modified Kubelka-Munk model. Linear regression for the determination of the bandgap energy is shown. Materials: (a) Degussa P25; (b) Anatase; (c) Acid synthesis TiO_2_. (**Right**) Diffuse refractance spectra for the studied TiO_2_ catalysts. (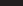) Degussa P25; (•••) Anatase; (-•-•-) Acid synthesis TiO_2_.

In order to establish further observations regarding the photocatalytic performance of the synthesized catalysts, their bandgap energy (Eg) was determined through diffuse refractance measurements. Figure 2 show diffuse refractance data plotted against the energy of the incident light, according to the Tauc modified Kubelka-Munk model (see methods for details). The values of Eg obtained for the synthesized catalyst (2.97 eV) are marginally lower than those determined for their commercial counterparts, with 3.03 eV and 3.17 eV for Degussa P25 and Anatase, respectively. The Eg results are in good agreement with the XRD data, particularly for the correspondence between AS-TiO_2_ and Anatase structures. Considering that the energy of the bandgap for AS-TiO_2_ is the lowest for the set of catalysts studied, and that the spectral response in the 250–400 nm range (Figure 2), between these catalysts is quite similar, it might be valid to expect for AS-TiO_2_ to be particularly efficient when using white light in the photocatalytic process, by requiring less energy for the valency band electrons to transition to the conduction band of the catalyst (UV/Vis spectra of RH6G as well as UV and White light radiant emission spectra are available in Supplementary Figure 2).

**Figure 3:**
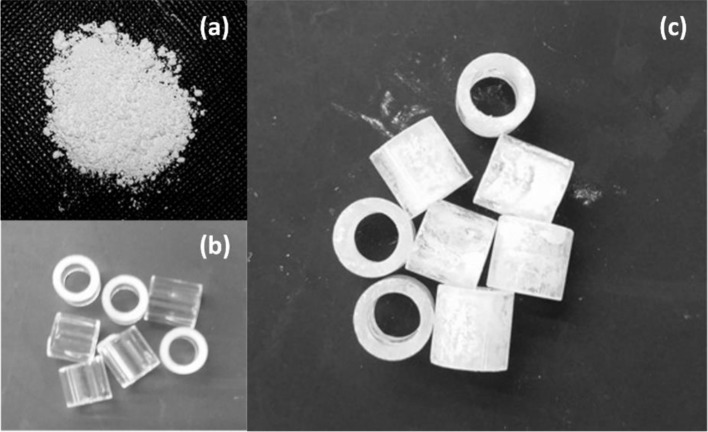
**(a)** Synthesized TiO_2_ catalyst. **(b)** Raschig rings without treatment. **(c)** Treated Raschig rings displaying supported TiO_2_ catalyst.

The synthesized TiO_2_ catalysts (Figure 3a), as well as commercial TiO_2_ samples (Degussa P25 and Anatase), all of them in the form a finely divided powder, were supported on Raschig rings (RR), made up of borosilicate glass (Figure 3b) of 5.0 mm of height, with 4.0 and 5.0 mm of internal and external diameter, respectively. The supporting procedure was based the solvent evaporation of a TiO_2_ suspension containing PEGMA-treated raschig rings, leading to the formation of an stable homogeneous layer of TiO_2_ on both the external and internal surfaces of the rings, as shown in Figure 3c.

The TiO_2_ support took place with varied efficiencies (Table 1). An important difference is observed on the support efficiency (SE) between AS-TiO_2_ (8% SE) and the commercial TiO_2_ samples. Degussa P-25 with a 20% SE and Anatase displaying the highest SE (26%). The observed differences in SE reported point toward raschig rings TiO_2_-loading variations based on granularity differences of the supported material, where the packing of the commercial TiO_2_ samples differs to that synthesized material, leading to the formation of layer(s) of different density between the samples studied.

**Table 1:** Support efficiency data for the thermal deposition of TiO_2_ catalysts on borosilicate Raschig rings (Refer to methods section for further details).

**TiO_**2**_ catalyst**	**Support efficiency (%)**
Degussa P25	20.1
Anatase	26.1
TiO_2_ (acid synthesis)	8.3

**Figure 4:**
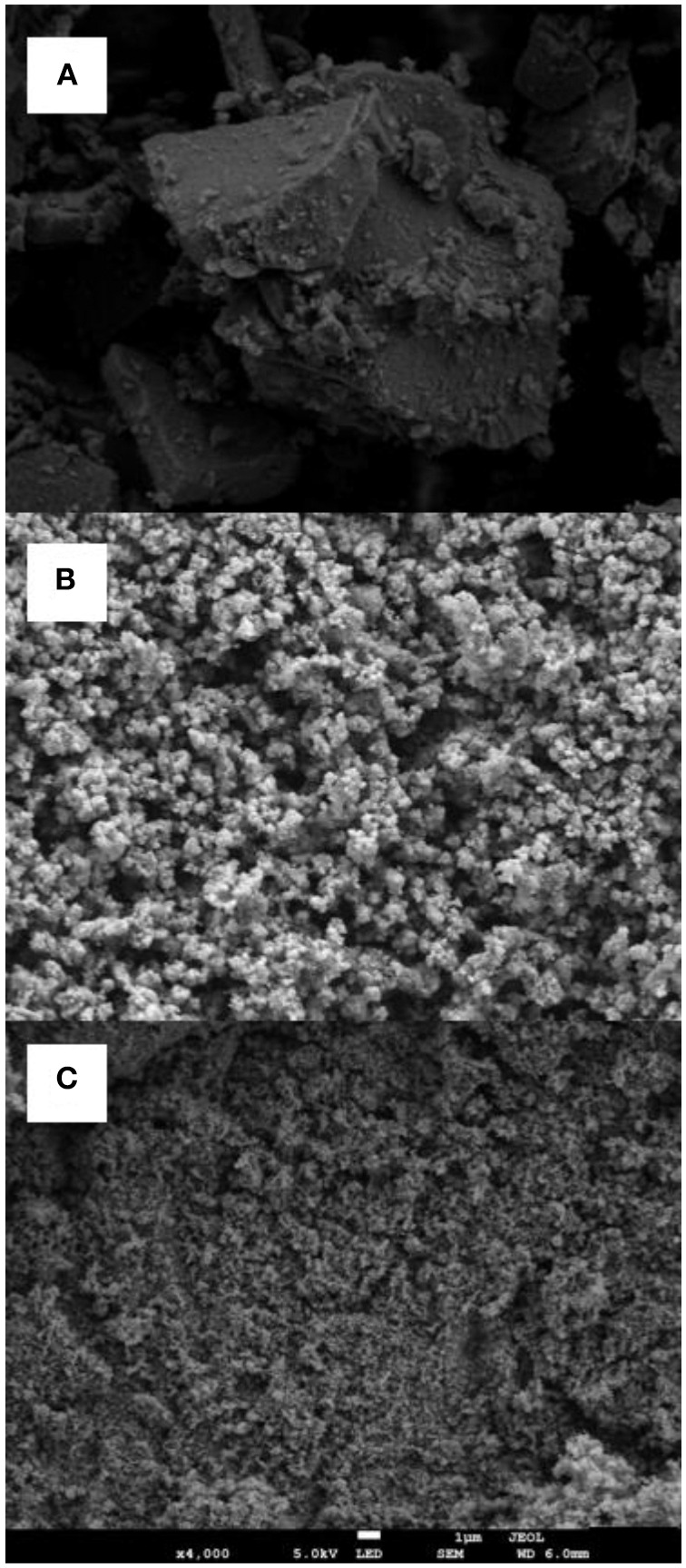
Scanning electron microscopy characterization of the studied TiO_2_ photocatalysts: **(A)** Acid synthesis TiO_2_; **(B)** Anatase; **(C)** Degussa. Magnification: 4000X, Beam energy: 5,0 kV.

In order to further support the claims regarding the differences in SE between the different TiO_2_ samples, physcochemical characterization of the materials were performed. SEM images reveal relevant differences between the considered TiO_2_ photocatalysts. AS-TiO_2_ samples (Figure 4A) display defined crystalline microparticulated aggregates formed by nanometric clusters which are identifiable at larger magnifications (>30 k X; See Supplementary Figure 3). On the contrary both Anatase (Figure 4B) and Degussa P25 (Figure 4C) present a more disperse configuration, without the evidence of important cluster formation or identifiable discrete crystalline formations of micrometric dimensions. From N_2_ adsorption/desorption isotherms evaluated under the BET model considerations, data shows (Table 2) that both AS-TiO_2_ and Anatase share a similar adsorption performance with similar specific adsorption area, in the range from 60 to 65 m^2^ per gram of adsorbent, whereas Degussa P25 shows a lower specific area, below 50 m^2^ per gram of adsorbent. Regarding the porosity of the systems, AS-TiO_2_ average pore size locates the material closer to the micro-mesoporous regime. Anatase on the other hand, operates on the mesoporous range, with an average pore size that is twice as large as that of AS-TiO_2_ and nearly half of the average pore size determined for Degussa P25 (nearly 40 nm) indicating that the porosity regime of the latter leans toward the mesoporous/macroporous range.

**Table 2:** Specific surface area (S_BET_) of the studied TiO_2_ photocatalysts, according to the Brunnauer, Emmet and Teller mode.

**Sample**	**S_**BET**_ (m^**2**^/g)**	**Pore diameter (nm)**
AS-TiO_2_	60.5	7.1
Anatase	65.9	18.9
Degussa P25	49.0	43.4

*Textural identity is expressed as average pore diameter of the samples*.

### Photodegradation of Rhodamin 6G by Raschig Rings-Supported TiO_2_

The photostability of Rh6G was studied in homogenous media by irradiating aqueous solutions of Rh6G using different light sources. The experimental configuration for the irradiation of the aqueous solutions of Rh6G, in the absence and the presence of supported catalysts, comprises a vertically-oriented sealed borosilicate glass tube located in a photoreactor with a series of lamps (UV or white light) lined up equally at each side of the tube (see Supplementary Figure 4), in order to achieve a total homogenous irradiation of the samples. Rh6G degradation kinetic data and degradation efficiency are shown in Table 3. The decrease of Rh6G concentration vs. time data behaved in accord to the pseudo-first order treatment (monoexponential decay), according to the Langmuir-Hinshelwood model for kinetics in solid-liquid interfaces and the kinetic profiles are shown in Supplementary Figure 5 of this work.

**Table 3:** Kinetic data for the photodegradation of Rh6G in homogeneous media and in the presence of supported TiO_2_ catalysts.

**Light source/catalyst^**a**^**	**k_**obs**_ (min^**−1**^)**	**Degradation percentage**
UV (365 nm)	1.7 × 10^−2^ ± 2.9 × 10^−3^	21.9 ± 3.2
UV (365 nm)/Degussa P25	2.1 × 10^−2^ ± 6.7 × 10^−3^	57.3 ± 6.8
UV (365 nm)/Anatase	2.2 × 10^−2^ ± 1.4 × 10^−2^	75.1 ± 3.0
UV (365 nm)/TiO_2_ acid synthesis	2.5 × 10^−2^ ± 9.0 × 10^−3^	77.5 ± 7.8
White light	2.0 × 10^−2^ ± 5.6 × 10^−3^	22.9 ± 4.3
White light/Degussa P25	4.0 × 10^−2^ ± 7.8 × 10^−3^	27.1 ± 4.7
White light/Anatase	2.6 × 10^−2^ ± 3.1 × 10^−3^	36.8 ± 5.2
White light/TiO_2_ acid synthesis	3.1 × 10^−2^ ± 4.5 × 10^−3^	66.8 ± 11.9

a *Experiments performed using 10 TiO_2_-loaded Raschig rings*.

When UV light (365 nm) is used to irradiate a solution of Rh6G a degradation percentage of 22% is determined, efficiency that is further increased in the presence of supported TiO_2_ catalysts, with a near three-fold increase for Degussa P25 and a four-fold increase for Anatase and AS-TiO_2_ with Rh6G degradation efficiencies above 70%. Similarly, the degradation rate is improved in the presence of the supported catalysts, with AS-TiO_2_ displaying the highest degradation rate.

The degradation efficiency of Rh6G in solutions irradiated with white light (23%) is quite similar to that observed with UV light irradiated samples (22%). For the supported catalysts, particularly for Degussa P25 and Anatase, the degradation efficiency observed is lower (compared to UV light) when white light is used to irradiate the samples, with no significative differences between the degradation of Rh6G in homogeneous media compared to heterogeneous media using Degussa P25 as catalyst. Similarly, only a moderate enhancement of the degradation efficiency is observed when Anatase is used. Interestingly, the performance of the AS-TiO_2_ supported catalyst is particularly good when irradiated with white light, inducing a three-fold increase on the degradation efficiency of Rh6G, increase that is only marginally lower than that observed with UV light.

Regarding the degradation rates observed for the white light irradiated samples, the analysis is not as straightforward as for UV light, with all of the catalysts moderately increasing the rate over the value observed in homogeneous media, with Degussa P25 doubling the degradation rate, followed by AS-TiO_2_, and Anatase having almost no difference with the rate determined in homogenous media. In homogeneous media, namely in the absence of TiO_2_, Rh6G absorbs light, undergoing a transition to its triplet state, in which this state Rh6G can release the energy and return to its ground state, get decomposed by photolysis or it can react with O_2_ and generate singlet oxygen (^1^O_2_), which then can react with Rh6G leading to the formation of oxidation products. In the presence of the catalysts, the process becomes more complex, with the dye-mediated sensitization process and the intrinsic ability of TiO_2_ working in tandem to generate ROS, which then readily contributes toward the degradation of Rh6G, further potentiating the photocatalytic process. From this, some observations can be made from the data in Table 2:

- In the absence of TiO_2_, degradation rates are similar irrespective of the light source used, indicating that the decomposition of Rh6G observed takes place by photolysis of the dye rather than ROS-induced oxidation.
- When UV light is used the degradation of the dye takes place mainly by ROS generated due to the intrinsic photocatalytic activity of TiO_2_ and by that induced by photosensitization, in all the TiO_2_ variants studied.
- The synthesized TiO_2_ catalytic performance is slightly superior to its commercial counterpart (Anatase) when UV light is used, but greatly improves when visible light is used, this is due mainly by the decrease on the Eg of the synthesized catalyst (compared to Anatase), reduced photolysis of the dye, as well the enhancement of the photosensitization process.

In a comparison between the performance of the supported TiO_2_ catalysts, and an equivalent mass of TiO_2_ catalyst in suspension, it is revealed that an important fraction of the photodegradation efficiency is preserved in the supported material, going from ~80 to 90% of photodegradation for all the TiO_2_ suspensions (Table 4) to a 77% (UV light) and 66% (white light) as maximum values of photodegradation (Table 3) achieved by the supported materials (AS-TiO_2_ and Anatase). The main cause of the observed differences might be associated to a loss of effective area of the supported TiO_2_ catalysts due to adsorption on the rings surface vs. the total surface availability of the suspended particles of TiO_2_. The percentage of retained photocatalytic activity in the supported TiO_2_ catalyst is particularly good, particularly when taking into account the large surface area of TiO_2_ catalysts lost upon support on the raschig rings, fact that is especially evident for the AS-TiO_2_, material which displays the lower support efficiency and morphologically differs the most from the others TiO_2_ variants (Anatase and Degusa P25), as presented in Figure 4.

**Table 4:** Kinetic data for the photodegradation of Rh6G on TiO_2_ catalysts aqueous suspensions irradiated with UV light.

**Catalyst^**a**^**	**k_**obs**_ (min^**−1**^)**	**Degradation percentage**
Degussa P25	6.5 × 10^−2^	80
Anatase	3.6 × 10^−1^	89
TiO_2_ acid synthesis	1.0 × 10^−1^	91

a *Mass of suspended catalyst is determined as the equivalent mass of supported TiO_2_ on 10 raschig rings*.

In order to delve further in to the photocatalytic processes of the systems studied, the chemical oxygen demand (COD) for the photocatalyzed decomposition Rh6G was evaluated. The determination of the COD of the photocatalytic process can provide useful information in these type of complex oxidative systems where parallel and sequential reactions might take place, systems in which common first order kinetics or initial rate kinetic methods might be somewhat limited to describe the overall process, particularly when spectrophotometric methods are used and the oxidative bleaching of the target molecule may not fully correspond with the whole oxidative pathway of said molecule (Mills et al., 2012). Similarly, COD has been successfully used in comparative studies involving semiconductor photocatalysts (El-Mekkawi et al., 2016). Data in Table 5 shows COD values and Rh6D photodegradation percentages for the studied photocatalysts. The minor differences observed in the degradation percentages determined by COD in all of the supported TiO_2_ variants, indicate that, besides the different complex photophysical processes taking place in various degrees, potentially involving different oxidation intermediaries, the endproducts of the considered photocatalytic systems appears to be similar.

**Table 5:** Chemical oxygen demand (COD) data for the photocatalytic degradation of Rhodamin 6G in the presence of supported TiO_2_ catalysts irradiated with UV light.

**Catalyst**	**COD (mg/L)**	**Degradation (%)**
Degussa P25	20	25
Anatase	21	33
TiO_2_ acid synthesis	21	33

### Influence of Additives on the TiO_2_-Catalyzed Photodegradation of Rhodamin 6G

The presence of additives and the study of their influence on the photocatalytic process is a matter of utmost importance, especially when considering the prospect of technological applications of the supported TiO_2_ catalyst on waste water treatment, were salinity and the presence of oxidant agents may affect the performance of the catalysts. With this purpose, the addition of NaCl, Na_2_SO_4_, and H_2_O_2_ to the Rh6G solutions was considered, and their influence on the AS-TiO_2_ photocatalytic activity was assessed (kinetic profiles in Supplementary Figures 6, 7). Data on Table 6 shows that for H_2_O_2_ there is a decrease in the photodegradation efficiency (60%) compared to that in the absence of the additive (77%; Table 3) when UV light is used. Similarly, an almost equivalent decrease is observed when white light is used, with a 52 vs. 67% (Table 3) with and without H_2_O_2_ respectively. On the other hand, for NaCl, and for both of the light sources studied, a negligible decrease of the photodegradation efficiency is observed, with a 72% degradation with UV light and a 62 % for white light. As observed with NaCl, the addition of Na_2_SO_4_ induces no significative variation into the photodegradation efficiency observed with white light, contrary to a larger decrease when UV light is used, with a 62 and a 61% photodegradation with white light and UV light, respectively.

**Table 6:** Influence of additives on the kinetic data (21 °C) for the photodegradation of Rhodamin 6G in the presence of TiO_2_ (acid synthesis) supported on Raschig rings.

**Additive**	**k_**obs**_ (min^**−1**^)**	**Degradation percentage**
**UV light (365 nm)**		
H_2_O_2_ (5 μM)	1.9 × 10^−2^ ± 3.0 × 10^−3^	60.1 ± 1.3
NaCl (5 μM)	1.9 × 10^−2^ ± 4.0 × 10^−3^	72.0 ± 2.6
Na_2_SO_4_ (5 μM)	1.0 × 10^−2^ ± 1.9 × 10^−3^	61.4 ± 8.8
NaCl (5 μM)/H_2_O_2_ (5 μM)	2.5 × 10^−2^ ± 3.5 × 10^−3^	63.3 ± 1.9
Na_2_SO_4_ (5 μM)/H_2_O_2_ (5 μM)	2.4 × 10^−2^ ± 1.0 × 10^−3^	68.6 ± 1.6
**White light**		
H_2_O_2_ (5 μM)	1.7 × 10^−2^ ± 4.0 × 10^−3^	51.6 ± 0.3
NaCl (5 μM)	2.7 × 10^−2^ ± 4.2 × 10^−3^	64.3 ± 6.9
Na_2_SO_4_ (5 μM)	2.9 × 10^−2^ ± 4.0 × 10^−3^	61.8 ± 5.6
NaCl (5 μM)/H_2_O_2_ (5 μM)	1.8 × 10^−2^ ± 8.0 × 10^−3^	55.4 ± 6.1
Na_2_SO_4_ (5 μM)/H_2_O_2_ (5 μM)	2.5 × 10^−2^ ± 8.1 × 10^−3^	59.3 ± 10.7

The kinetic data for the addition of H_2_O_2_ show a decrease on the rate constant (2.5 × 10^−2^ min^−1^ without H_2_O_2_; Table 3) for both light sources, whereas for ${\text{SO}}_{4}^{-2}$ and Cl^−^ sodium salts there is no change in the rate constants when white light is used, but decreases when the samples are irradiated with UV light, with the largest decrease being observed for ${\text{SO}}_{4}^{-2}$ (1.0 × 10^−2^ min^−1^; Table 6).

Overall, the data presented regarding the influence of the ${\text{SO}}_{4}^{-2}$, Cl^−^ and hydrogen peroxide, shows a good tolerance of the photocatalytic system to these additives. The explanation for the observed influence of the anions in the system can be complex, for example both anions can interact with the vacant holes of the generated in the valence band, inhibiting the recombination of the hole/electron pair on the photocatalysts surface as well as to react with H_2_O, generating hydroxyl radicals, leading to further degradation of Rh6G, however it has been also reported that these ions can also display an inhibitory behavior (Yan et al., 2012), by scavenging hydroxyl radicals generated on the surface of the catalyst, as well as by competing with Rh6G for the adsorption sites available, which seem to be the most likely explanation on the behavior of the data presented for the photodegradation of Rh6G in the studied system.

For the case of hydrogen peroxide, at nanomolar concentrations (Sahel et al., 2016; Kang et al., 2017), it can act as a scavenger of electrons from the conduction band of TiO_2_, which promotes charge separation and the formation of hydroxyl radicals, which may lead to an enhanced degradation of Rh6G. However, a decrease of photodegradation efficiency is observed in our system, indicating that at the considered H_2_O_2_ concentrations, electron and/or hydroxyl radical scavenging properties of hydrogen peroxide might be of relevance.

When equimolar ratios of H_2_O_2_ and salt are used, interesting results are observed. First, when UV light is used, the rate for the mixture NaCl/H_2_O_2_ is equal to that observed without additives, and higher than the values determined for the systems containing NaCl and H_2_O_2_ separately. On the contrary, for the samples irradiated with white light, the mixture shows a rate constant equal to that of the system containing only hydrogen peroxide, lower than the one determined in the absence of additives. The behavior observed for the samples containing the mixture Na_2_SO_4_/H_2_O_2_ is quite similar, for UV light, the rate of the photocatalytic process taking place in the presence of both ${\text{SO}}_{4}^{-2}$ and H_2_O_2_ is higher than that of the additives separately, and equal to that observed in the absence of additives, the same being true for white light, but the rate of the mixture is now equal to that observed when Na_2_SO_4_ is the only additive present in the photocatalytic system.

## Conclusions

Raschig rings-supported TiO_2_ catalysts display a good photocatalytic performance when compared to equivalent amounts of TiO_2_ in aqueous suspension, even though a large surface area of TiO_2_ material is lost upon support. The comparative study between suspension vs. supported TiO_2_ catalysts reveals that optimization of the available area in the raschig rings is imperative in order to improve the catalyzed photodegradation of Rh6G. This is particularly evident by taking into account that the characteristics (XRD, RD, Eg) and observed photodegradative performance of the synthesized catalysts are in good agreement with the commercial TiO_2_ samples, and that the Rh6G photodegradation differences observed with the light sources considered are minimal in the presence of TiO_2_ catalysts.

The presence of additives induce changes in the kinetics and efficiency of the TiO_2_-catalyzed photodegradation of Rh6G, particularly when white light is used in the process, pointing toward a complex phenomenon, however the stability of the supported photocatalytic systems is acceptable in the presence of the studied additives. In line with this, the magnitude of the chemical oxygen demand, indicates that besides the different complex photophysical processes taking place, the intermediate products of the considered photocatalytic systems appears to be similar.

## Data Availability Statement

The raw data supporting the conclusions of this article will be made available by the authors, without undue reservation, to any qualified researcher.

## Author Contributions

EP designed the experiments, analyzed the results, and wrote and revised the manuscript. CC analyzed the results and wrote and revised the manuscript. FH and GA performed experimental activities. GC participated in the data analysis and discussions. All authors have approved the final revised manuscript.

## Conflict of Interest

The authors declare that the research was conducted in the absence of any commercial or financial relationships that could be construed as a potential conflict of interest.
