# Supplementary material for: Photocatalytic Degradation of Aqueous Rhodamine 6G Using Supported TiO2 Catalysts. A Model for the Removal of Organic Contaminants From Aqueous Samples
Source: Front Chem. 2020 May 5;8:365. doi: 10.3389/fchem.2020.00365 (PMC7215082; doi:10.3389/fchem.2020.00365)
Supplement: Supplementary file 1 [file Table_1.DOCX]

Electronic supplementary material for

“Photocatalytic degradation of aqueous Rhodamine 6G using supported TiO_2_ catalysts. A model for the removal of organic contaminants from aqueous samples”

**Table of contents**

| **Data** | **Page** |
| --- | --- |
| **Figure SM1.** XRD diffraction pattern for Degussa P-25 TiO2 catalyst. Data obtained using a Bruker D8 advanced difractometer with a Pure Cu-Kα1 parallel beam. | **3** |
| **Figure SM2.** UV/Vis Absorption spectra of Rhodamine 6G **(▬)**; UV light emission spectra **(▬);** white light emission spectra **(▬).** | **3** |
| **Figure SM3.** Scanning electron microscopy characterization of acid synthesis TiO_2_ photocatalyst. Magnification: 30000X, Beam energy: 5,0 kV. | **4** |
| **Figure SM4.** A) Configuration of the sample inside the photoreactor for the irradiation procedures. B) Sample tube containing Rhodamin 6G and the TiO_2_-loaded Raschig rings. C) UV light irradiation of the sample, taking place inside the photoreactor. | **5** |
| **Figure SM5.** Rh6G (5 uM) degradation for the TiO_2_ catalysts studied, irradiated with diferent light sources. A) UV (365 nm), B) White light. TiO_2_ samples: Degussa P25 (■), anatasa (●), acid synthesis TiO_2_ (▲). Inset show linearized pseudo first oder tendencies of the kinetic data. | **6** |
| Figure SM6. Rh6G (5 μM) photostability in absence (■) and presence of additives: (○) H_2_O_2_ 5 μM (Δ) NaCl 5 μM, (◊) Na_2_SO_4_ 5 μM, (▼) NaCl 5 μM y H_2_O_2_ 5 μM, (►) Na_2_SO_4_ 5 μM y H_2_O_2_ 5 μM. Light sources: con A) UV light (365 nm), B) White light. | **7** |
| **Figure SM7.** Rh6G (5 uM) degradation using A) UV light and B) white light, in the presence of additives, using acid synthesis TiO2 as catalyst. :(□) H_2_O_2_ 5 μM (●) NaCl 5 μM, (Δ) Na_2_SO_4_ 5 μM, (▼) NaCl 5 μM y H_2_O_2_ 5 μM, (♦) Na_2_SO_4_ 5 μM y H_2_O_2_ 5 μM. | **8** |

**Figure SM1.** XRD diffraction pattern for Degussa P-25 TiO2 catalyst. Data obtained using a Bruker D8 advanced difractometer with a Pure Cu-Kα1 parallel beam.

**Figure SM2.** UV/Vis Absorption spectra of Rhodamine 6G **(▬)**; UV light emission spectra **(▬);** white light emission spectra **(▬).**


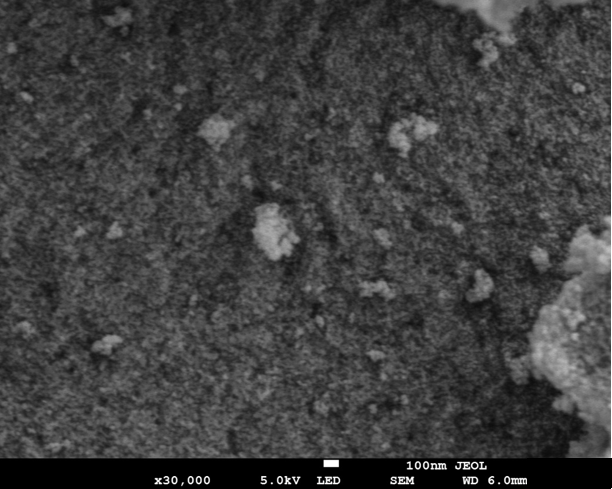


**Figure SM3.** Scanning electron microscopy characterization of acid synthesis TiO_2_ photocatalyst. Magnification: 30000X, Beam energy: 5,0 kV.

**
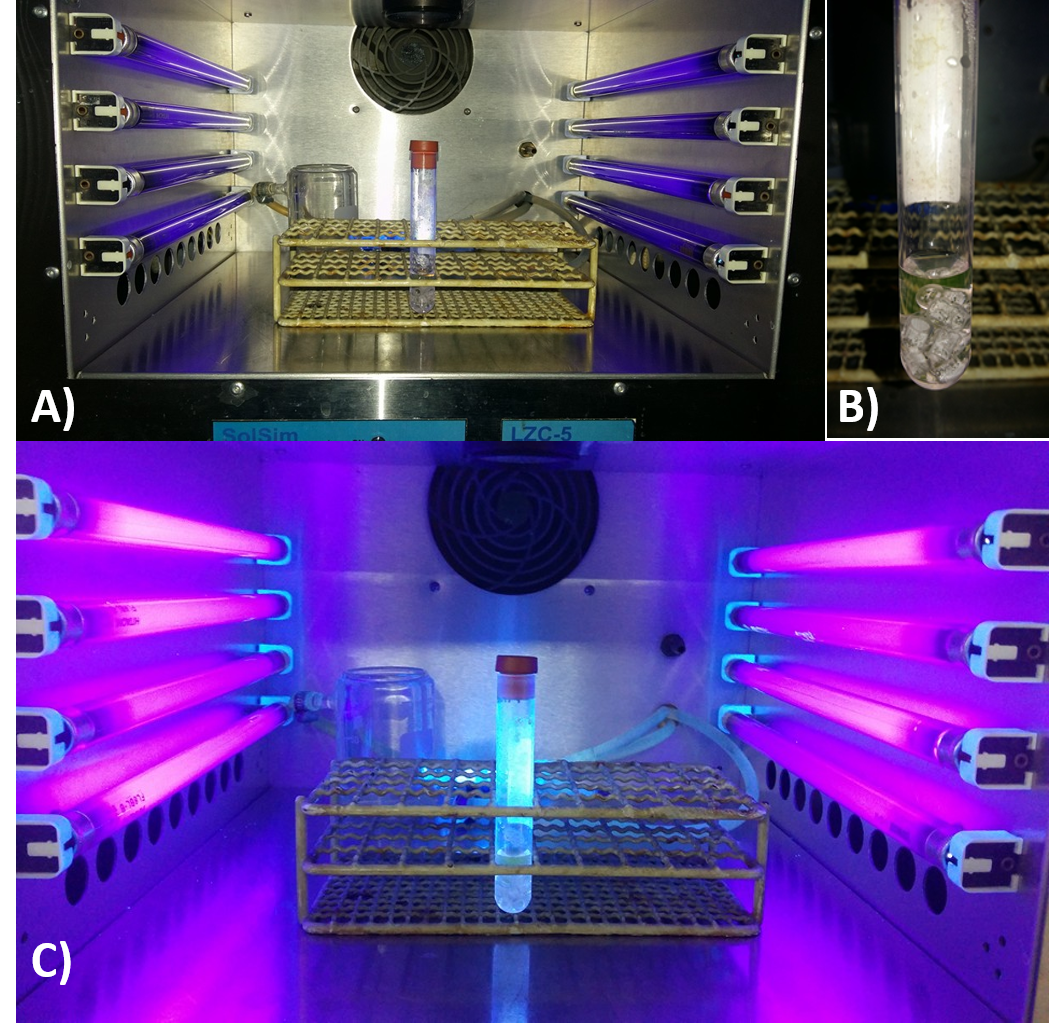
**

**Figure SM4.** A) Configuration of the sample inside the photoreactor for the irradiation procedures. B) Sample tube containing Rhodamin 6G and the TiO_2_-loaded Raschig rings. C) UV light irradiation of the sample, taking place inside the photoreactor.

**Figure SM5.** Rh6G (5 uM) degradation for the TiO_2_ catalysts studied, irradiated with different light sources. A) UV (365 nm), B) White light. TiO_2_ samples: Degussa P25 (■), Anatase (●), acid synthesis TiO_2_ (▲). Inset show linearized pseudo first order trends of the kinetic data.

Figure SM6. Rh6G (5 μM) photostability in absence (■) and presence of additives: (○) H_2_O_2_ 5 μM (Δ) NaCl 5 μM, (◊) Na_2_SO_4_ 5 μM, (▼) NaCl 5 μM y H_2_O_2_ 5 μM, (►) Na_2_SO_4_ 5 μM y H_2_O_2_ 5 μM. Light sources: con A) UV light (365 nm), B) White light.

**Figure SM7.** Rh6G (5 uM) degradation using A) UV light and B) white light, in the presence of additives, using acid synthesis TiO_2_ as catalyst. :(□) H_2_O_2_ 5 μM (●) NaCl 5 μM, (Δ) Na_2_SO_4_ 5 μM, (▼) NaCl 5 μM y H_2_O_2_ 5 μM, (♦) Na_2_SO_4_ 5 μM y H_2_O_2_ 5 μM.
